# Systematic versus sentinel-lymph-node-driven axillary-lymph-node dissection in clinically node-negative patients with operable breast cancer. Results of the GF-GS01 randomized trial

**DOI:** 10.1007/s10549-018-4733-y

**Published:** 2018-03-10

**Authors:** P. Roy, A. Leizorovicz, R. Villet, C. Mercier, J. Y. Bobin

**Affiliations:** 1 Service de Biostatistique-Bioinformatique, Hospices Civils de Lyon, 162 Avenue Lacassagne, 69003 Lyon, France; 2 Université de Lyon, Lyon, France; 3 Université Lyon 1, Villeurbanne, France; 4 Laboratoire de Biométrie et Biologie Évolutive, CNRS, UMR 5558, Villeurbanne, France; 5 Service de pharmacologie clinique et essais thérapeutiques, Hospices Civils de Lyon, Bron, France; 6 Service de chirurgie viscérale et gynécologique, Groupe hospitalier Diaconesses-Croix-Saint-Simon, Paris, France; 7 Service de Chirurgie Oncologique, Hospices Civils de Lyon, Pierre-Bénite, France

**Keywords:** Breast cancer, Clinical trial, Sentinel-lymph-node biopsy, Surgery, Survival

## Abstract

**Purpose:**

Sentinel-lymph-node (SLN) resection seems to minimize systematic axillary-lymph-node dissection (sALND) side effects in operated breast cancer patients. We explored whether SLN resection achieves similar therapeutic outcomes as sALND but with fewer side effects.

**Methods:**

A randomized, controlled, open-label trial with parallel-group design compared sALND restricted to cases with positive SLN biopsy (test arm, *n* = 774) versus SLN biopsy followed by sALND (control arm, *n* = 770).

**Results:**

The five-year overall survivals in control and test arms were 96.42 and 95.64% (*P* = 0.2925). The estimated difference was nearly zero (precisely, − 0.79%, one-tailed 95% confidence interval (CI) limit − 2.44%). In a multivariate Cox model, the adjusted hazard ratio in the test arm was HR 0.81 (upper 95% CI limit 1.17). Advanced age (HR 1.05 per additional year, CI [1.03–1.08]), negative progesterone receptor (HR 2.17 [1.35–3.45]), SLN metastasis (HR 1.69 [1.03–2.79]), and only one SLN identification technique (HR 4.14 [1.21–14.18]) were associated with lower survival. Patients with ≥ 1 severe side effect at 1 month in control and test arms were 173/703 = 24.6% [21.5–28.0%] and 91/693 = 13.1% [10.7–15.9%] (*P* < 0.001). The estimated sensitivity of SLN biopsy (control arm) was 145/178 = 81.5% [74.8–86.7%].

**Conclusions:**

Restricting ALND to cases with positive SLN biopsy does not affect the overall survival but reduces by 11.5% [7.5–15.6%] (*P* < 0.001) the risk of severe short-time side effects of sALND.

## Introduction

Systematic axillary-lymph-node dissection (ALND) is a procedure aimed to establish nodal status and guide adjuvant treatment indication to maximize survival and regional control of cancer in breast cancer patients. However, this procedure has been shown associated with short-term and long-term side effects in a substantial number of patients [1–3]. Sentinel-lymph-node (SLN) resection was proposed to minimize ALND side effects [4]. Within this context, the GF-GS01 trial was designed to establish whether SLN resection achieves similar therapeutic outcomes as ALND but with fewer side effects. The initial aim of the GF-GS01 trial was to demonstrate the non-inferiority of the test arm (ALND restricted to positive SLN) in terms of relapse-free survival. The trial expected fewer post-surgical complications.

## Methods

### Trial design

GF-GS01 is a randomized, controlled, open-label trial with a parallel-group design (ClinicalTrials.gov Identifier: NCT00144898). SLN resection was performed in both arms, whereas ALND was systematic in the control arm but restricted to SLN-positive patients in the test arm.

### Participants

Patients were included in 70 centers in France between August 2003 and June 2007. To be eligible, women aged 18 or older had to present invasive breast cancer ≤ 30 mm at clinical examination or mammography, confirmed by needle (cytology) or micro/macro biopsy (histology), without clinical node involvement (N0) or organ metastasis (M0). An informed written consent was obtained from each participant.

### Axillary sentinel-lymph-node identification

At least 25 min before entrance to the operating room, the patient was given an injection of one mCi of Technetium-99 m colloidal rhenium sulfide (1 mL followed by injection of 0.2 mL of air) intradermally for superficial tumors or in the gland close to the tumor for deep tumors. Injecting into the tumor was strictly forbidden. The skin was marked when radioactive lymph nodes were localized using a gamma probe, the arm being positioned in surgical posture. Patent blue (Gerbet, France) was injected in the operating room in the presence of an anesthetist, either a single intradermal injection of two mL in front of the tumor or two peritumoral injections of one mL each. Lymph nodes that were radioactive and/or blue were labeled as sentinel lymph nodes. An extemporaneous examination of the sentinel lymph node was performed only in case of suspicion of nodal involvement by the surgeon or the pathologist. Optionally, the internal mammary chain sentinel-lymph node could also be explored in the case of internal or medial tumors. Lymphoscintigraphy was optional and not subject to evaluation in this trial. Sentinel-lymph nodes were identified before being sent to the pathologist.

SLNs without detectable metastasis on paraffin stains [Hematoxylin and Eosin stains (HE), Hematin, Phloxine, and Saffron stains (HPS)] including serial node sectioning were analyzed using immunohistochemical (IHC) techniques. Non-SLNs were analyzed with HPS, completed with IHC techniques when a doubt persisted after HPS examination.

### Number of subjects needed (initial calculation)

Under the alternative hypothesis of identical disease-free survival probabilities, 382 events (relapse or death) had to be observed to reject the null hypothesis of a hazard ratio of 1.35 in 90% of the studies (*β* = 10%), a type-one error rate *α* = 5% (one-tailed) being retained. On the basis of a planned accrual period of 2 years, a 5-year follow-up of the last patient included, and an expected 5-year disease-free survival of 85% in the control arm, it was decided to randomize 2152 patients (1076 per arm). Under the hypothesis of detection of sentinel lymph node in 95% of the patients and a discovery of a metastatic SLN on extemporaneous examination in 5% of cases, 2400 patients had to be included in the trial.

### Randomization

When SLN was not macroscopically suspect on biopsy, the patients were randomly assigned to “test” and “control” arms in a 1:1 ratio (centralized computer randomization). Randomization was stratified on age at study entry (≤ 50, > 50 years) and study center. Because masking was not possible due to the nature of the procedures, the co-investigators had no information about the randomization process (mixture of blocks of various sizes).

### Follow-up

Patients were planned to be monitored for overall survival, disease-free survival, and regional cancer control up to 5 years. An administrative request was made to obtain the official vital status of women at end of follow-up.

### Outcomes

The initial primary endpoint of the trial was relapse-free survival. Secondary endpoints included sentinel false-negative rate (control arm), post-surgical complications during the first month and later, and overall survival. The first month post-surgical complications included axillary infection, axillary lymphedema, axillary hematoma, axillary bleeding, axillary paresthesia or intercostobrachial nerve injury, pain with arm movement, brachial plexus injury, and “other” complications.

The trial had to overcome several difficulties, i.e., lack of financial resources, decrease of investigators motivation, whereas there was no evidence of non-inferiority of the test arm in the literature. The follow-up of patients was not completed in several centers. Then, neither the primary endpoint nor the late side effects were available for the majority of the patients. At the blind review, the steering committee decided to change the primary endpoint into overall survival and restrict the analysis of the secondary endpoints to those available at 1 month of follow-up (i.e., the analysis of relapse-free survival was dropped). It was also decided to exclude seven centers (83 patients) because of unavailable follow-up data.

### Sample size

The number of patients of the GF-GS01 non-inferiority trial was initially calculated for the disease-free survival endpoint. According to the steering committee, there was no justification for a new power calculation on the new primary endpoint after the closure of the trial.

### Statistical analysis

No interim endpoint analyses were planned. The analyses were carried out according to the intent-to-treat principle.

Overall survival was analyzed as primary outcome. The follow-up of still-alive patients was censored at the date of the vital status request (June 18, 2012). Data on patients lost to follow-up were censored at the date of last follow-up. The survival curves were estimated using Kaplan–Meier methods [5] and compared using the log-rank test. The estimated difference in the probability of 5-year survival was calculated together with the corresponding lower limit of the one-tailed 95% confidence interval (CI). A frailty proportional hazard regression model [6, 7] was fitted, with “center” as random effect. Survivals of patients randomized in the test and the control arms were compared using Wald test and the estimated hazard ratio (HR) (test arm versus control arm) with its 95% CI upper limit.

A secondary analysis of survival was performed including, in the model, variables “treatment arm” and “age”, and testing the following variables: pathological tumor size, Scarff-Bloom-Richardson (SBR) grade, SLN status, Body Mass Index (BMI), tumor location, histological type, hormone receptors, metastatic embolization, SLN identification procedure, and non-surgical treatments (radiotherapy, adjuvant chemotherapy, adjuvant hormone therapy). The assumption of proportional hazard was checked by analyzing Schoenfeld residuals. A *P* value < 5% in Wald test was considered for statistical significance.

Post-surgical complications at 1 month were described by arm and compared using Fisher exact tests. The occurrence of at least one complication was compared between test and control arms by fitting a mixed effects unconditional logistic regression model, with a fixed effect put on variable “arm”, random effects put on variable “center”, and “arm-center” interaction. This model included variable “age” and tested variables “BMI” and “history of shoulder disease”.

The proportions of patients with positive SLN were estimated in each arm (with the corresponding 95% CI) and compared using a Fisher exact test.

In the control arm, the factors associated with axillary-lymph-node involvement and SLN involvement were analyzed fitting unconditional logistic regression models that included systematically “age” (as fixed effect) and “center” (as random effect). The analysis investigated the proportion of false negatives, i.e., the proportion of patients with negative SLN among those with positive ALND. Candidate risk factors for a false-negative result were analyzed. The type-1 error rate was fixed at *α* = 0.05 in all analyses.

The analyses were performed with SAS/STAT software, version 9.1.3 for Windows and the survival package of R software, version 2.13.0 (http://www.r-project.org/).

## Results

### Participants flow

**Fig. 1:**
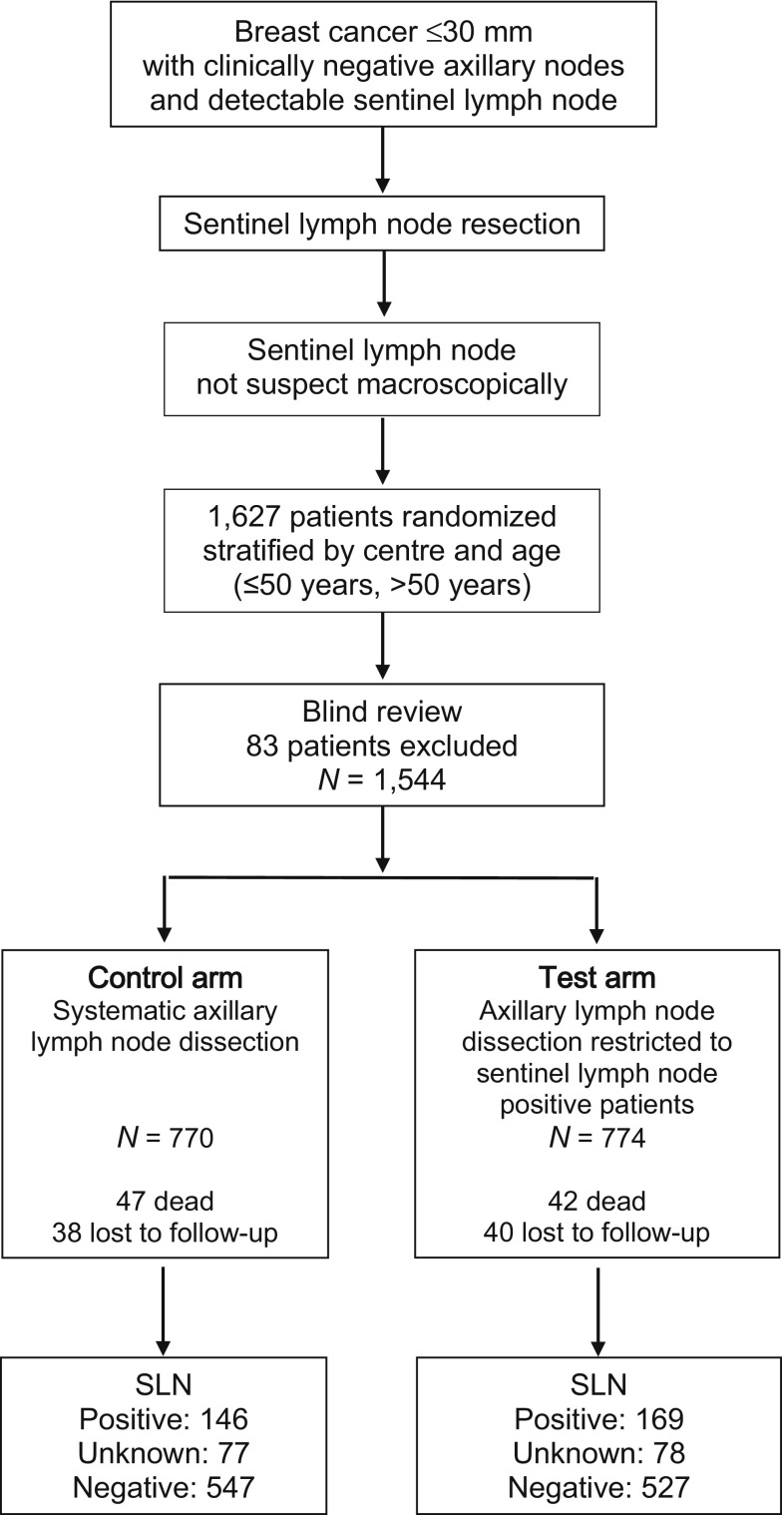
Flow chart of GF-GS01 trial

Figure 1 presents a description of the trial. The study randomized 1627 women. After exclusion of 83 patients from seven centers, 1544 patients were left for the statistical analysis: 770 in the control arm and 774 in the test arm. Protocol violations included non-compliance with eligibility criteria (five patients in the control arm vs. six in the test arm), disagreement between randomization and actual axilla treatment (one vs. four patients, respectively), and stratification error (13 vs. 12 patients, respectively).

### Baseline data

Patient and tumor characteristics at inclusion are shown in Table 1. The distributions of the baseline characteristic were similar in the two arms. In all patients, history of shoulder disease was reported by 2.7% of the patients and the WHO performance status was excellent in 84.1%. Technetium-99 m colloidal rhenium sulfide plus patent blue dye plus lymphoscintigraphy were used for axillary sentinel-lymph-node detection in 84.9% of all cases. In 81.6% of all cases, the tumor did not exceed 2 cm on pathology laboratory examination. Receptors for estrogen and progesterone were positive in 87 and 75% of all tumors, respectively.

**Table 1:** Patient characteristics at baseline

Variable at inclusion and modality	Systematic ALND	ALND restricted to positive SLN	Total
	*N* = 770	*N* = 774	*N* = 1544
Patient characteristics			
Age	59.2 ± 10.9 (696)	59.6 ± 11.2 (693)	59.4 ± 11.0 (1389)
Body Mass Index	24.9 ± 4.5 (670)	24.6 ± 4.3 (673)	24.8 ± 4.4 (1343)
History of shoulder disease	20/706 (2.8%)	18/700 (2.6%)	38/1406 (2.7%)
History of familial breast cancer	76/704 (10.8)	91/703 (12.9)	167/1407 (11.9)
History of familial ovary cancer	11/703 (1.6)	6/703 (0.9)	17/1406 (1.2)
Hormone replacement therapy	84/699 (12.0)	89/695 (12.8)	173/1394 (12.4)
WHO performance status/*n*	/663	/663	/1326
0	563 (84.9)	552 (83.3)	1115 (84.1)
1	83 (12.5)	93 (14.0)	176 (13.3)
2	11 (1.7)	9 (1.4)	20 (1.5)
3	4 (0.6)	1 (0.2)	5 (0.4)
4	2 (0.3)	8 (1.2)	10 (0.8)
Tumor clinical presentation			
Breast side	/706	/704	/1410
Right	331 (46.9)	335 (47.6)	666 (47.2)
Left	375 (53.1)	369 (52.4)	744 (52.8)
Breast clinical aspect/*n*	/697	/694	/1391
Normal	550 (78.9)	539 (77.7)	1089 (78.3)
Adipose	104 (14.9)	115 (16.6)	219 (15.7)
Fibrocystic mastopathy	39 (5.6)	33 (4.8)	72 (5.2)
Other	4 (0.6)	7 (1.0)	11 (0.8)
Tumor location/*n*	/705	/705	/1410
Upper-outer	311 (44.1)	348 (49.4)	659 (46.7)
Lower-outer	98 (13.9)	75 (10.6)	173 (12.3)
Upper-inner	139 (19.7)	118 (16.7)	257 (18.2)
Lower-inner	48 (6.8)	63 (8.9)	111 (7.9)
Several/other	109 (15.5)	101 (14.3)	210 (14.9)
Clinical tumor size/*n*	/699	/703	/1402
T0	166 (23.7)	170 (24.2)	336 (24.0)
T1	439 (62.8)	454 (64.6)	893 (63.7)
T2	92 (13.2)	77 (11.0)	169 (12.1)
Other	2 (0.3)	2 (0.3)	4 (0.3)
Tumor preoperative management			
Mammography			
Stellar lesion	315/572 (55.1)	296/562 (52.7)	611/1134 (53.9)
Nodular lesion	297/561 (52.9)	291/560 (52.0)	588/1121 (52.5)
Microcalcifications	94/498 (18.9)	90/504 (17.9)	184/1002 (18.4)
Architectural distortion	79/493 (16.0)	112/511 (21.9)	191/1004 (19.0)
Other	21/443 (4.7)	20/445 (4.5)	41/888 (4.6)
Localization by harpoon	220/692 (31.8)	219/690 (31.7)	439/1382 (31.8)
Micro/macro biopsy	567/692 (81.9)	534/685 (78.0)	1101/1377 (80.0)
Cytology	201/663 (30.3)	202/652 (31.0)	403/1315 (30.6)
Primitive tumor management			
Type of surgery	/697	/698	/1395
Radical mastectomy	15 (2.2)	15 (2.1)	30 (2.2)
Lumpectomy	682 (97.8)	683 (97.9)	1,365 (97.8)
Pathological staging	/688	/692	/1380
Microinvasive	4 (0.6)	5 (0.7)	9 (0.7)
pT1a	44 (6.4)	42 (6.1)	86 (6.2)
pT1b	183 (26.6)	198 (28.6)	381 (27.6)
pT1c	327 (47.5)	323 (46.7)	650 (47.1)
pT2 < 3 cm	102 (14.8)	101 (14.6)	203 (14.7)
pT2 > 3 cm	13 (1.9)	14 (2.0)	27 (2.0)
Others	15 (2.2)	9 (1.3)	24 (1.7)
SBR	/663	/671	/1334
I	196 (29.6)	223 (33.2)	419 (31.4)
II	345 (52.0)	326 (48.6)	671 (50.3)
III	122 (18.4)	122 (18.2)	244 (18.3)
Estrogen receptors (IHC)	/683	/675	/1358
Positive	596 (87.3)	587 (87.0)	1183 (87.1)
Negative	87 (12.7)	88 (13.0)	175 (12.9)
Progesterone receptors (IHC)	/679	/673	/1352
Positive	510 (75.1)	503 (74.7)	1013 (74.9)
Negative	169 (24.9)	170 (25.3)	339 (25.1)
SLN technique	/701	/699	/1400
Tec or PB	9 (1.3)	13 (1.9)	22 (1.6)
(Tec or PB) + LS	45 (6.4)	46 (6.5)	91 (6.5)
Tec + PB	47 (6.7)	51 (7.3)	98 (7.0)
Tec + PB + LS	600 (85.6)	589 (84.3)	1189 (84.9)

Results are expressed as mean ± SD (*n*), numerator/denominator (*n*), or *n* (%)

*ALND* axillary-lymph-node dissection, *SBR* Scarff, Bloom, and Richardson grading system, *IHC* immunohistochemistry, *SLN* sentinel lymph node, *Tec* technetium-99m, *PB* patent blue, *LS* lymphoscintigraphy

### Main endpoint

**Fig. 2:**
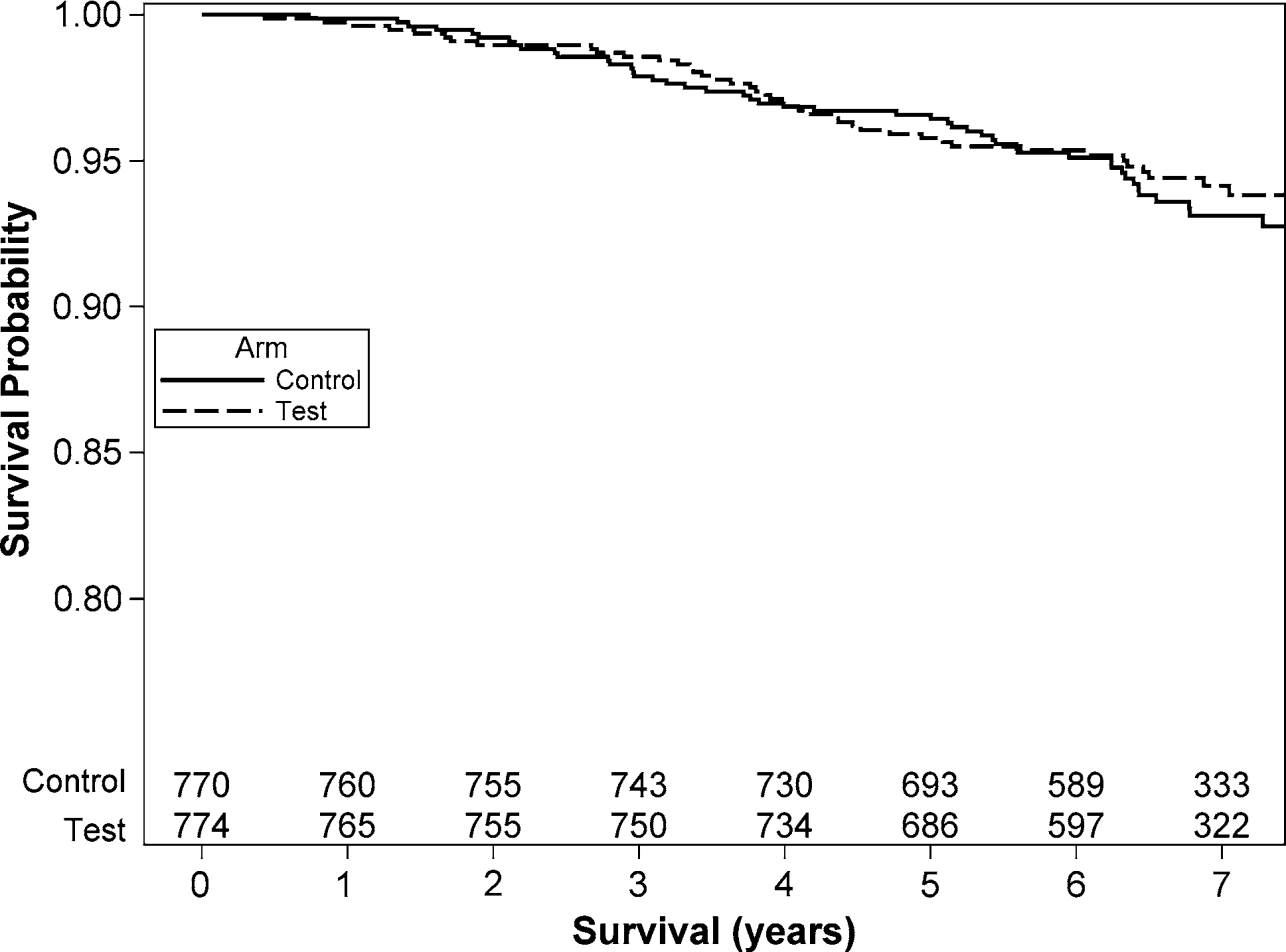
Overall survival curves for test and control arms

At the cut-off date (June 18, 2012), 89 patients were deceased and 78 lost to follow-up. The estimated overall survival probabilities were similar in the two study arms. Figure 2 shows that Kaplan–Meier overall survival curves superimpose. The estimated 5-year overall survival was 96.42% (95% CI 95–98%) in the control arm versus 95.64% (95% CI 94–97%) in the test arm. The null hypothesis of identical survivals between the two treatment arms could not be rejected (log-rank Chi-square test = 0.2983, one-tailed *P* value = 0.2925). The estimated 5-year difference in survival probability (test arm minus control arm) was − 0.79%, and the corresponding lower limit of the corresponding one-tailed 95% CI was − 2.44%. When a frailty proportional hazard regression model adjusted on age and center (random effect) was fitted, the hazard ratio for the test arm was HR 0.91 with an upper 95% CI limit (one-tailed) of 1.31.

In the secondary analysis of survival, the independent prognostic factors associated with mortality were advanced age (HR 1.05 per additional year, 95% CI 1.03–1.08), negative test for progesterone receptor (HR 2.17, 95% CI 1.35–3.45), SLN metastasis (HR 1.69, 95% CI 1.03–2.79), and the use of only one axillary sentinel-lymph-node identification technique (HR 4.14, 95% CI 1.21–14.18). Factor Scarff-Bloom-Richardson grade III (HR 1.69, 95% CI 0.99–2.87) was close to statistical significance. The adjusted hazard ratio for the test arm was 0.81 with an upper 95% CI limit (one-tailed) of 1.17 (Table 2).

**Table 2:** Results of the multivariate analysis. Hazard ratios for each explanatory variable modality

Variable and modality	*N*	HR (95% CI)^a^	*P* value^b^
Age (years)	1364	1.05 (1.03–1.08)	< 0.001
Age²	1364	1.00 (1.00–1.00)	0.127
SBR			0.054
I–II	1124	1	
III	240	1.69 (0.99–2.87)	
Progesterone receptors			0.001
Positive	1024	1	
Negative	340	2.17 (1.35–3.45)	
SLN metastasis			0.039
Negative	1053	1	
Positive	311	1.69 (1.03–2.79)	
SLN identification			0.024
(Tec + PB) ± LS	1346	1	
Only Tec or PB	18	4.14 (1.21–14.18)	
Arm			0.173
Control	682	1	
Test	682	0.81 (NA–1.17)	

*HR* hazard ratio, *SBR* Scarff, Bloom, and Richardson grade, *Tec* technetium-99m, *PB* patent blue, *SLN* sentinel lymph node, *LS* lymphoscintigraphy

^a^One-tailed 95% CI for clinical trial arm, two-tailed 95% CI for other variables

^b^Wald Test. One-tailed for clinical trial arm, two-tailed for other variables

### Post-surgical complications at one month

The prevalence of short-term complications was significantly lower in the test arm than in the control arm (Table 3). The estimated absolute differences in prevalence between the control and the test arm were 12.1% (95% CI 8.1–16.1%) for axilla seroma, 17.6% (95% CI 13.2–22.0%) for axillary paresthesia or intercostobrachial nerve injury, and 13.9% (95% CI 9.6–18.2%) for pain on arm movement. The proportion of patients presenting at least one severe side effect at 1 month was 173/703 = 24.6% (95% CI 21.5–28.0%) in the control arm versus and 91/693 = 13.1% (95% CI 10.7–15.9%) in the test arm; i.e., the estimated difference in prevalence was 11.5% (95% CI 7.5–15.6%, *P* < 0.001). The proportion of patients presenting at least one side effect at 1 month was 405/703 = 57.6% (95% CI 54–61%) in the control arm versus 224/693 = 32.3% (95% CI 29–36%) in the test arm (*P* < 0.001). When a mixed effects unconditional logistic regression model was fitted, neither BMI nor history of shoulder disease was retained as independent risk factors of presenting at least one complication. Between-center heterogeneity led to introduce an arm-center interaction term. The prevalence of patients presenting at least one side effect at 1 month decreased with age (OR 0.99 per additional year, 95% CI 0.98–1.00) and was importantly reduced in the tested arm (mean OR 0.30, 95% CI 0.22–0.42, between-center 95% credible interval: 0.16–0.62).

**Table 3:** Comparison of the prevalence of post-surgical complications at 1 month between test and control arm

First month side effects	Systematic ALND	ALND restricted to positive SLN	Prevalence difference (95% CI)	*P* value^a^
	*N* = 703	*N* = 693		
Axillary infection	11 (1.6%)	8 (1.2%)		0.645
Axillary seroma	159 (22.6%)	73 (10.5%)	12.1% (8.1–16.1)	< 0.001
Axillary hematoma	12 (1.7%)	17 (2.5%)		0.354
Axillary bleeding	1 (0.1%)	1 (0.1%)		1.000
Axillary paresthesia or intercostobrachial nerve injury	218 (31%)	93 (13.4%)	17.6% (13.2–22.0)	< 0.001
Pain on arm movement	192 (27.3%)	93 (13.4%)	13.9% (9.6–18.2)	< 0.001
Serratus anterior nerve injury	0 (0%)	1 (0.1%)		0.497
Other complications	38 (5.4%)	33 (4.8%)		0.627
Severe short-time side effects (≥ 1)	173 (24.6%)	91 (13.1%)	11.5% (7.5–15.6)	< 0.001
Short-time side effect (≥ 1)	405 (57.6%)	224 (32.3%)	25.3% (20.1–30.5)	< 0.001

*ALN* axillary lymph nodes (sentinel LN plus others), *ALND* axillary-lymph-node dissection, *SLN* sentinel lymph node

^a^Two-tailed Fisher exact test

### Proportions of patients with positive sentinel-lymph node

The prevalence of positive SLN in all patients was 315/1389 = 22.7% (95% CI 20.5–25.0%); 146/693 = 21.1% in the control arm; and 169/696 = 24.3% in the test arm. The estimated difference (3.2%) was not significant with 95% CI − 1.3 to + 7.8% (*P* = 0.172).

### Probability of positive axillary lymph node and positive SLN (control arm)

Positive axillary node (SLN or non-SLN) were observed in 180/680 = 26.5% (95% CI 23.2–30.0%) in the control arm. One SLN-positive patient had a non-SLN unknown status and one non-SLN-positive patient had an SLN unknown status.

The probability of positive axillary lymph node increased together with tumor size (OR 1.93 per additional cm, 95% CI 1.53–2.44) and negativity of tumor estrogen receptors (OR 2.18, 95% CI 1.16–4.08) but decreased together with age at diagnosis (OR 0.98 per additional year, 95% CI 0.96–0.99) and inner location of the tumors, particularly upper-inner tumor (OR 0.49, 95% CI 0.29–0.83) (Table 4). Similar results were observed when factors associated with sentinel-lymph-node involvement were analyzed (Table 4).

**Table 4:** Multivariate analysis of factors associated with axillary-lymph-node involvement and sentinel-lymph-node involvement (control arm)

Variable	ALN involvement			SLN involvement		
	*N*	OR (95% CI)^a^	*P* ^b^	*N*	OR (95% CI)^a^	*P* ^b^
Age (year)	668	0.98 (0.96–0.99)	0.009	667	0.97 (0.95–0.99)	0.003
Tumor size (cm)	668	1.93 (1.53–2.44)	< 0.001	667	1.72 (1.35–2.19)	< 0.001
Tumor location						
Upper-outer	291	1		291	1	
Lower-outer	95	1.38 (0.82–2.30)	0.226	95	1.13 (0.65–1.96)	0.675
Upper-inner	134	0.49 (0.29–0.83)	0.008	133	0.48 (0.27–0.86)	0.013
Lower-inner	47	0.54 (0.24–1.26)	0.154	47	0.73 (0.31–1.68)	0.453
Several/other	101	0.69 (0.40–1.21)	0.198	101	0.71 (0.40–1.28)	0.259
Estrogen receptors						
Positive	585	1		584	1	
Negative	83	2.18 (1.16–4.08)	0.016	83	2.11 (1.08–4.15)	0.030

*ALN* axillary lymph nodes (sentinel plus others), *SLN* sentinel lymph nodes

^a^Two-tailed 95% CI

^b^Wald test

### Probability of false-negative SLN (control arm)

SLN and non-SLN were both positive in 41 patients and both negative in 499 patients, whereas 104 patients were SLN positive/non-SLN negative, and 33 patients were SLN negative/non-SLN positive. The estimated sensitivity of the SLN was 145/178 = 81.5% (95% CI 74.8–86.7%), and the corresponding probability of false-negative result was estimated at 33/178 = 18.5% (95% CI 13.3–25.2%). No significant risk factor was associated with the probability of a false negative, whereas a non-significant trend was observed for larger tumors and lower-outer locations (Table 5).

**Table 5:** Factors associated with false-negative sentinel-lymph-node results

Variable	*N*	OR (95% CI)^a^	*P*
Age	178	1.02 (0.98–1.06)	0.275
Tumor size (cm)	178	1.55^b^ (0.93–2.59)	0.099
Tumor location			
Upper-outer	87	1	
Lower-outer	34	2.14 (0.84–5.47)	0.114
Upper-inner	24	1.02 (0.35–3.02)	0.968
Several/other	33	0.94 (0.29–3.08)	0.921

^a^Two-tailed 95% CI

^b^Per additional cm

## Discussion

In operable breast cancer patients, the present phase III trial compared axillary-lymph-node dissection restricted to cases with sentinel lymph node (SLN) positive versus sentinel lymph node plus systematic axillary-lymph-node dissection (control arm) in terms of overall survival (primary endpoint) and post-surgical complications (secondary endpoints).

When the trial was initiated, the issue was important and few randomized clinical trials were designed to provide an answer. Overall survival was not considered to be different between the two arms; indeed, the hazard ratio for the test arm was 0.91 (unadjusted) with an upper 95% CI limit of 1.31, and 0.81 (adjusted) with an upper 95% CI limit of 1.17. Similar results were observed in two other randomized trials designed to answer the same question. In the Milanese trial [8], 516 patients were randomized according to the present trial design but the major endpoint was the occurrence of axillary metastasis. The Milanese trial reported 38 deaths and a 10-year overall survival of 93.5% (95% CI 90.3–96.8%) in the test arm versus 89.7% (95% CI 85.5–93.8%) in the control arm (log-rank test, *P* = 0.15). In the USA/Canadian NSABP B-32 trial [9], 309 deaths were reported among 3986 women with follow-up information; the estimated 5-year and 8-year overall survivals were 95.0 and 90.3%, respectively, in the test arm versus 96.4 and 91.8% in the control arm, and the estimated unadjusted hazard ratio was 1.2 (95% CI 0.96–1.50, *P* = 0.12). The design of a third trial, Almanac (UK) [10], was close to the present one; 1031 patients were randomized into two arms (a test arm = sentinel lymph node + axillary clearance or axillary radiotherapy in case of positive SLN and a control arm = SLN + systematic axillary-lymph-node dissection) and the follow-up was restricted to 18 months because the major aim of the trial was assessing the patients’ quality-of-life. In Almanac, at 12 months after surgery, seven deaths occurred in each arm.

Because of a less aggressive therapy in the test arm and the nature of the major endpoint criteria, a non-inferiority trial design was retained for the GF-GS01 trial, the Milanese trial, and the NSABP B-32 trial. One difficulty in such a trial design is providing a difference (or a ratio) to be rejected (which corresponds to rejection of the null hypothesis) or a threshold of equivalence for the difference in point estimate (or ratio). The Milanese trial was designed to reject a 5% difference or more in the proportion of axillary nodal metastases at 5 years. After a mean follow-up of 95 months, only 2 axillary metastases were observed, a much lower rate than expected, which did not allow a clear conclusion on the main endpoint of the trial [8]. The NSABP B-32 trial was designed to declare equivalence upon a 2% difference in survival or less between the two treatment arms among sentinel-node-negative patients [9]. Initially based on disease-free survival, the retained null hypothesis in the present GF-GS01 trial was a hazard ratio of 1.35 or more (See Number of subjects needed—initial calculation). In non-inferiority trials, the estimated upper limit of the 95% CI of the effect size is more informative than the level of significance. The estimated upper limits of the 95% CIs of the HRs of the GF-GS01 trial (1.17) and the NSABP B-32 trial (1.49) were almost close (note that the confidence intervals were one-sided in GF-GS01 trial and two-sided in NSABP B-32 trial).

A positive SLN was observed in 169/696 = 24.3% of the patients in the test arm versus 146/693 = 21.1% of the patients in the control arm. This positivity rate of SLN in the control arm was smaller than the one observed in the NSABP B-32 trial (694/2672 = 26.0% [11] or in the Milanese trial (83/257 = 32.3%) [12]; however, here, IHC techniques were systemically used in case of negative sentinel lymph nodes, whereas they were used only for confirmation of suspected metastases in the NSABP B-32 or the Milanese trial. This result is not surprising because, here, the sensitivity of the SNL was 81.5% and its specificity was 100% by construction [13]. The corresponding estimated false-negative rate was 33/178 = 18.5% (95% CI 13.3–25.2%), which is higher than the rates observed in the NSABP B-32 trial (75/766 = 9.8%) [11], the Milanese trial (8/91 = 8.8%) [12], or a meta-analysis (7.3%) performed on 7754 patients of whom 3132 had nodal involvement [14]. Here, it is interesting to note that, in 14 out of 33 false-negative cases, only one sentinel lymph node was resected and that the mean number of sentinel lymph nodes resected in the control arm (i.e., 2.2) is comprised between the mean numbers found in the Milanese trial (1.7) [12] and in the NSABP B-32 trial (2.9) [11].

The proportion of short-time side effects is substantially different between the control and the tested arm (57.6% vs. 32.3%, respectively), the estimated absolute difference in prevalence being 25.3% (95% CI 20.1–30.5%, *P* < 0.001). Differences in prevalence of axillary seroma (12.1%), axillary paresthesia or intercostobrachial nerve injury (17.6%), and pain on arm movement (13.9%) contributed greatly to this difference. A comparison regarding the prevalence of side effects between the 100 first consecutive patients included in each arm of the Milanese trial showed that axillary pain, paresthesias on the operated side, and alteration of arm mobility at 6 and 24 months were more frequent in the control than in the test arm [12]. In the NSABP B-32 trial, the 12-month ipsilateral arm symptom mean score was 3.6 in the control arm versus 2.5 in the test arm (*P* = 0.006) [15].

## Conclusions

In the field of breast cancer without clinical node involvement or organ metastasis, the GF-GS01 trial is the second randomized trial in terms of number of women included in a comparison of axillary-lymph-node dissection restricted to SLN-positive patients with SLN biopsy followed by systematic axillary-lymph-node dissection. This GF-GS01 trial confirms that the former procedure reduces the risk of severe short-time side effects attributable to systematic axillary dissection by 11.5%, without affecting the overall survival. Results from similar trials are welcome to provide a more accurate estimation of the effect size.

## Data availability

The data that support the findings of this study are not publicly available. They are available on request after the agreement of the study scientific board.

## Abbreviations

ALND: Axillary-lymph-node dissection
BMI: Body Mass Index
CI: Confidence interval
GF-GS01: Acronym of the trial
HE: Hematoxylin and eosin stains
HIC: Immunohistochemical
HPS: Hematin, phloxine, and saffron stains
HR: Hazard ratio
OR: Odds ratio
SBR: Scarff-Bloom-Richardson
SLN: Sentinel lymph node (n) or sentinel-lymph-node (comp. n. or adj.)
